# Durable anti-fog micro-nano structures fabricated by laser ablation of aluminum film on resin/glass

**DOI:** 10.1186/s11671-024-03993-y

**Published:** 2024-03-26

**Authors:** Hongtao Cui, Chao Teng, Xinyi Xie, Xiaowen Qi

**Affiliations:** Department of Materials Science, School of Civil Engineering, Qingdao University of Technology, Qingdao, 266520 China

**Keywords:** Superhydrophilic, Antifog, Laser ablation, Micro-nano structures, Durability

## Abstract

This study presents a technique for processing transparent glass and resin substrates using a low-cost laser marker to create a micro-nano-structured surface with exceptional anti-fog properties. The approach involved depositing an aluminum (Al) film on the transparent substrates as an absorbing layer, followed by rapid laser marker ablation. This ablation process effectively removed the majority of the Al film, resulting in the formation of hierarchical hillock-hollow micro-structures and the dispersion of Al-based nano-particles throughout the surface. The resulting structure on resin glasses demonstrated anti-fog performance even after 629 days storage in the laboratory, which marked the longest antifog record. It exhibited impressive antifog property without visible degradation for the first 9 months, which though degraded substantially afterwards. Furthermore, the micro-nano structure played a key role in reducing the contact angle of the surface. The contact angle experienced a significant reduction from a value of 64° for the control resin to 6.9° for the treated resin, while it was reduced from 44° for the control glass to 0° for the treated glass, indicating superhydrophilicity. This 0° superhydrophilic state persisted for a period of 25 days.

**Supplementary Information:**

The online version contains supplementary material available at 10.1186/s11671-024-03993-y.

## Introduction

Glass and resin are widely used in applications such as optical sensors, medical endoscopes, eyeglass lenses, and windshield glass due to their excellent optical properties [1, 2]. However, one common issue faced with these transparent materials is fogging [3], which can negatively impact their functionality. Fog or contaminants block the user’s vision, and prevent one from working correctly. Fogging on medical endoscope lenses during surgery can affect normal operation and even cause medical accidents, endangering lives [4, 5]. Another prevalent concern pertains to fogging on automotive glass during rainy day and cold winter, jeopardizing lives during drive. Fog-resistant technologies have received widespread attention in the fields of optical instruments [6], sensors [7], medical lenses [8], automotive glass, solar panels [8–10].

To address the problem of surface fogging, two main approaches have been employed. The first approach involves adjusting environmental parameters such as relative humidity and temperature to avoid the conditions required for fogging, including heating the substrate and increasing the air flow rate near the surface [11–13]. However, this method is energy-and-time consuming. The second approach focuses on modifying the surface properties of the transparent materials to either increase hydrophilicity or hydrophobicity. Super hydrophilicity and superhydrophobicity have garnered significant interest as they can mitigate the fogging effect. Superhydrophobic surface may be inclined to allow the droplets to roll to eliminate the fogging effect [9, 14, 15]. The bouncing-off effect due to droplets coalescence-induced surface energy to kinetic energy conversion also facilitates water departure from the horizontally placed superhydrophobic surface [16, 17]. Hydrophobic surfaces get wet eventually when exposed to fog or humid atmosphere because droplets of a size comparable to that of the surface features tends to grow within the texture rather than atop distroying superhydrophobicity [16], transitioning from a Cassie state to Wenzel state [6]. Small nano-structures are therefore preferred for sustaining superhydrophobity, while they may be eroded by outdoor wind and sand. Meanwhile, it is complex and also technically challenging to fabricate superhydrophobic surfaces [18]. On the other hand, water spreads on superhydrophilic surfaces promtly and forms a continuous complete water film, eliminating fogging without compromising transparency [9, 19]. And superhydrophilic surfaces was reported achieving effective fog prevention [20, 21]. Therefore, superhydrophilic surface preparation is a widely applied fog prevention method [22].

Creating durable anti-fog coatings with strong adhesion to the substrate is crucial. Some organic coatings, although inherently hydrophilic, dissolved in water and were not long-lasting for anti-fogging effect [23]. Researchers had explored the use of SiO_2_ and TiO_2_ coatings to enhance anti-fog properties [24, 25]. SiO_2_ is a hydrophilic material by nature. Liu et al. [26] combined the layer-by-layer assembly technique and template method to develop SiO_2_ anti-fog film consisting of raspberry-like nanospheres that can protect against fog for more than 20 days. He et al. [27] prepared an anti-fog film of SiO_2_ composed of mulberry-shaped nanospheres that could maintain antifog property for more than 30 days. Wang et al. [28] manufactured TiO_2_ coatings that maintained anti-fogging for about 10 days. Yang et al. [29] employed iron-doped titania to reduce the bandgap width to extend the spectral range for photogenerated carriers, which remained super hydrophilic for up to 60 days. UV irradiation-cured polymer-modified titania nanoparticle composites remained hydrophilic for up to 330 days, but the lowest contact angle was approximately 2.5° [30], and its antifog performance was not ideal. Yu et al. [31] prepared a zwitterionic and hydroxylated super hydrophilic surface on eyeglasses. The anti-fog effect of these glasses was evident for one month under laboratory conditions, but the antifog effect degraded dramatically after seven months. While some of these coatings maintain their anti-fog properties for short periods, insufficient durability and transmission loss remain challenges.

Single-scale structure features are not adequate to achieve long lasting superhydrophilicy due to adsorption of airborne organics after exposure to ambient air [32]. Nano-structures inhibit organic diffusion and micro-structures promote surface wetting [32]. Microstructures protect and support nanostructures instrumental to the durability [33], a hierarchical micro-nano structure is therefore needed.

Special lasers are required to directly process transparent materials such as glass or resin substrates [34, 35]. Ultrashort lasers offer a direct method for processing glass or resin through multiphoton absorption and are a good candidate for manufacturing micro-nano structures due to the flexible, non-contact, controllable, and substrate-independent characteristics [36]. However, they can also cause damage such as micro-explosions and micro-cracks to the substrate itself [37]. Femtosecond and picosecond lasers are expensive and have low energy density. Ablation of samples is inefficient and requires multiple pulses, thus slow and costly [38, 39]. Additionally, nanosecond laser ablation of titania coatings on glass surfaces reached a minimum contact angle of 4.7° at a fairly slow rate below 100 mm/min and the depth of the micro-structure was at micrometers level, suggesting possible damage [34]. Titania might be the issue as was not light absorbing. Thermal conductivity and capacity of resin is even lower than that of glass, which means even lower thermal damage threshold. Even less laser energy deposition into resin is required. To address these challenges, this study employs a laser marker to ablate absorbing Al coated glass/resin substrate for fabricating micro-nano structure on its surface. Al, known for its lightweight, high strength, corrosion resistance, and weldability, was used as the laser radiation absorber [40, 41]. Most of the laser energy was consumed by ablating the Al film with minimal remaining deposited into the substrate and would cause negligible damage to the substrate. This technique offers simplicity, ease of control, affordability, high energy density, and high efficiency. The laser ablated Al nanoparticles were highly reactive from plasma plume [42, 43], which may react with the substrate to form strong chemical bonding. Such bonding may contribute to the durabililty because strong inorganic bondings were found contributing to durable superhydrophilicity [44].

## Experimental

### Materials

Al sputter targets had a high purity of 99.9995% and dimensions of D60 × 5 mm, which were supplied by Zhongnuo Advanced Material Technology Co., Ltd. The resin substrates had a transmission of approximately 88% in the visible spectral range. The resin substrates were made of polyallyl diglycol carbonate (CR-39), from Jiangsu Kangmeida Optical Co., Ltd and had varying myopia degrees of 100°, 200°, 300°, and 400°, along with a refractive index of around 1.49. Soda lime glass substrates were supplied by China Yaohua Glass Group Co., Ltd.

### Methods

All the substrates were subjected to the supersonic bath cleaning procedure in the sequence of acetone, isopropanol, and DI water, for 5 min each to remove surface contaminants. The clean substrates were then dried using compressed dry air. The dry substrates were then transferred from a load lock into a sputter deposition chamber. The chamber was evacuated to a background pressure of 7 × 10^–4^ Pa. A DC power of 255 W was applied for sputtering the water-cooled Al target. The sputtering gas was 99.999% argon (Ar) with a controlled flow rate at 30 sccm, and the working pressure in the chamber was set at 1.6 Pa. The Al coating thickness was varied for comparison: 0.6 μm, 1.1 μm, 1.6 μm, 2.1 μm, and 2.6 μm.

**Fig. 1:**
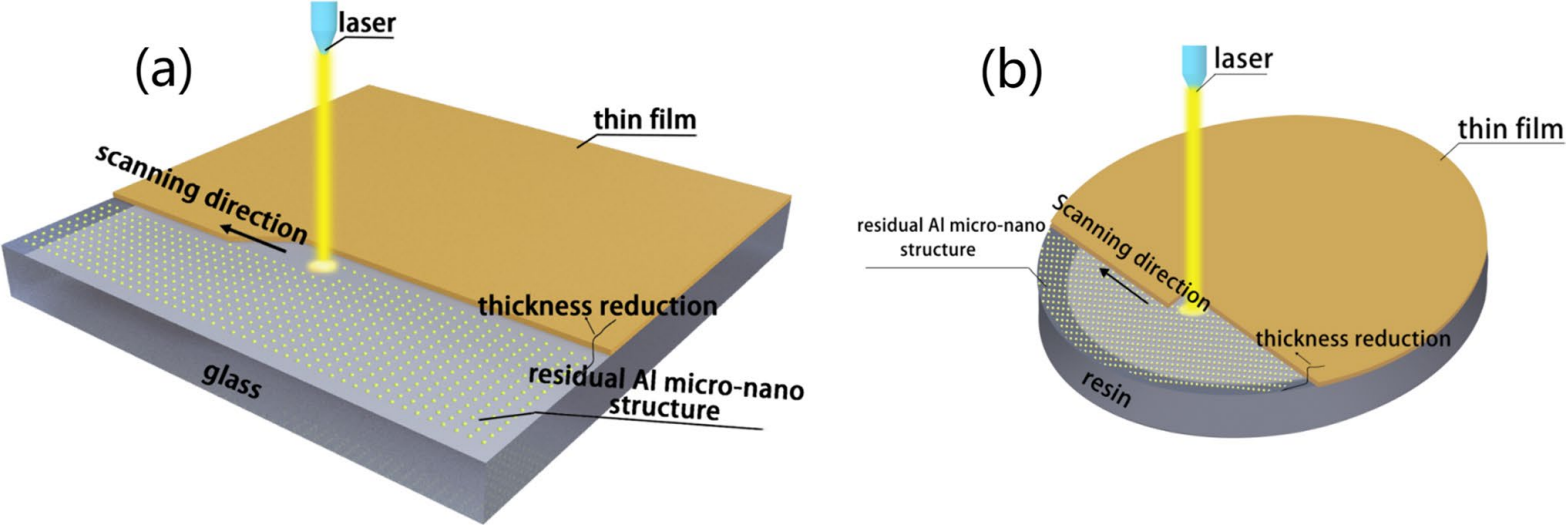
Schematic diagram of laser ablation of Al film on transparent substrate. **a** Glass. **b** Resin

A pulsed laser marker (YLP-M30) operating at a wavelength of 1064 nm, with a pulse duration of 100 ns, a repetition rate of 30 kHz and an output power of 9 W, was utilized to ablate the Al coated substrates. The laser beam was focused on the aluminum film with a focal spot diameter of 50 μm. The laser ablation was carried out in a line-by-line and cross-fill scan mode, with the line spacing of 0.02 mm. And the movement of the laser head controlled by a computer. The scanning rate was varied for comparison at 2 m/s, 3 m/s, 4 m/s, 5 m/s, and 6 m/s, the fluence was estimated to be 7.5–22.5 J/cm^2^ [45], respectively. The approach involved depositing an Al film on the transparent substrates as an laser absorbing layer, followed by rapid laser marker ablation. Figure 1 schematically demonstrates the laser ablation of Al film to create a micro-nano-structured surface.

### Characterization

Scanning electron microscope (SEM) images were obtained using the Sigma300 microscope from CARL ZEISS, which had a resolution of 1 nm. These images were used to extract the morphology of the laser-ablated surfaces. An Olympus OLS5100 laser scanning confocal microscope was utilized for surface morphology and roughness characterization. The 3D surface structure particularly data related to the depth dimension in nm level, was obtained using an atomic force microscope (AFM). AFM used in the study was Bruker Dimension Icon from Germany. Raman analyzer DXR12 with a laser excitation wavelength of 532 nm was used for structure characterization. The surface chemical compositions of the samples were analyzed using X-ray photoelectron spectroscopy (XPS, Thermo Scientific K-alpha). The goniometer SDC-100 from Dongguan, China was used for contact angle measurement, which was an indicator of surface wettability. A micro-pipette was used to dispense 1 µL of water droplets onto a sample surface for the measurement. A diffuser glass illuminated by an LED source was used as the background for imaging. A spectrophotometer Lambda 950 from Perkin Elmer was employed for transmission measurement in the wavelength range of 400-800 nm.

### Antifogging measurement

In the study, a boiling water vapor test was conducted to evaluate the antifogging performance of the samples. A hot water kettle was used to boil water, and the samples were positioned 40 mm above the kettle cover for 10 s individually. The anti-fogging performance of each sample was observed, compared, and photographed under a consistent background condition.

To assess the durability of the antifogging properties, multiple boiling water vapor tests were conducted spanning over several months. These tests were performed at different time intervals to evaluate the long-term effectiveness of the samples. The tests were conducted in real-time, with the background displaying the official website of the Greenwich Observatory showing Beijing time.

## Results and discussion

### Microstructure characterization

**Fig. 2:**
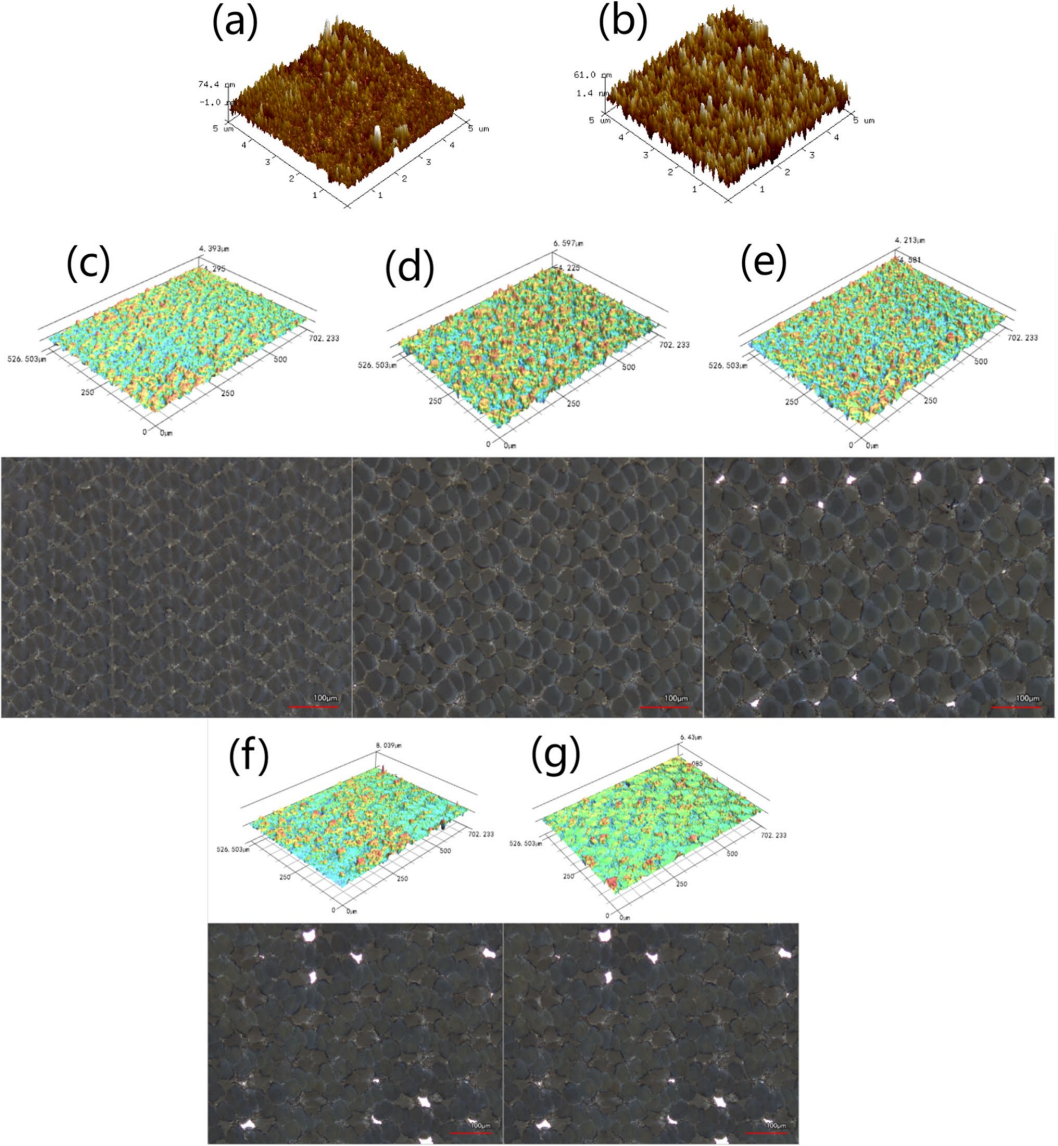
AFM images of laser ablated samples on different substrates with Al thickness of 1.6 μm and scan rate of 3 m/s. **a** Resin. **b** Glass. Confocal images of laser ablated Al-coated resin with varying scanning rate at the same 1.6 μm Al coating. **c** 2 m/s **d** 3 m/s, **e** 4 m/s, **f** 5 m/s, **g** 6 m/s

Figure 2 presents nano-scale and micro-scale surface morphology and roughness of treated resin and glass. Figure 2a–b shows the 3D morphologies of treated resin and glass under the same processing parameters, which exhibited similar characteristics. The surface of treated resin was relatively smooth, with a roughness (Ra) value of 9 nm. The treated glass displayed a slightly higher surface roughness with an Ra value of 13 nm. According to the Wenzel model, an increase in surface roughness enhances the hydrophilicity of the surface [46]. According to Young’s equation, higher surface energy surface leads to lower contact angle and better hydrophililcity [47]. Surface energy of glass was higher than that of resin. Therefore, treated glass tended to be more hydrophilic than treated resin.

The micro-scale surface morphology and roughness of treated samples was examined by laser scanning confocal microscopy, and the results were presented in Fig. 2c–g, and Table 1. Figure 2c–g depict the presence of hillock and hollow structures characterized by shallow depressions surrounded by narrow protrusions. These micro-structures were a result of laser scan overlapping, which can be estimated by laser focal spot diameter, line spacing, scan speed, and repetition rate and the spot to spot seperation was listed in Table 1 [48, 49]. The light colour regions in 2D images were non-overlapping regions. The lighter the more residuals. Figure 2c indicates 2 m/s led to least residuals in 2D image and presents large irregular exposed resin spots in 3D image, which suggest excessive ablation causing non-uniformity in the height dimension. Other than this, it was observed that both the non-uniformity and size of the features increased with higher scan speed. A scan rate of 3 m/s exhibited superior feature uniformity and the highest number of features. As indicated in Table 1, the surface roughness Ra reached its maximum value of 0.598 μm at 3 m/s. According to the Wenzel model, Ra plays a crucial role in determining the hydrophilicity of samples, implying that the sample treated at 3 m/s exhibited the most favorable hydrophilicity. The density and uniformity of the features also contribute to the creation of regular channels for efficient water spreading, thereby enhancing the hydrophilicity of the surface. In comparison with Fig. 2d, g presents that too high laser scanning speed caused underablation with too many residuals, led to loosely arranged surface microstructures lacking regularity, which was adverse to hydrophilicity. It is worth noting that both glass and resin substrates, treated under the same parameters, exhibited similar surface structures.

**Table 1:** The surface roughness (Ra) of the samples with varying scanning rate and the same 1.6 μm Al coating

	2 m/s	3 m/s	4 m/s	5 m/s	6 m/s
Surface roughness(μm)	0.304	0.598	0.469	0.311	0.231
Spot-to-spot separation(mm)	0.067	0.1	0.133	0.167	0.2

**Fig. 3:**
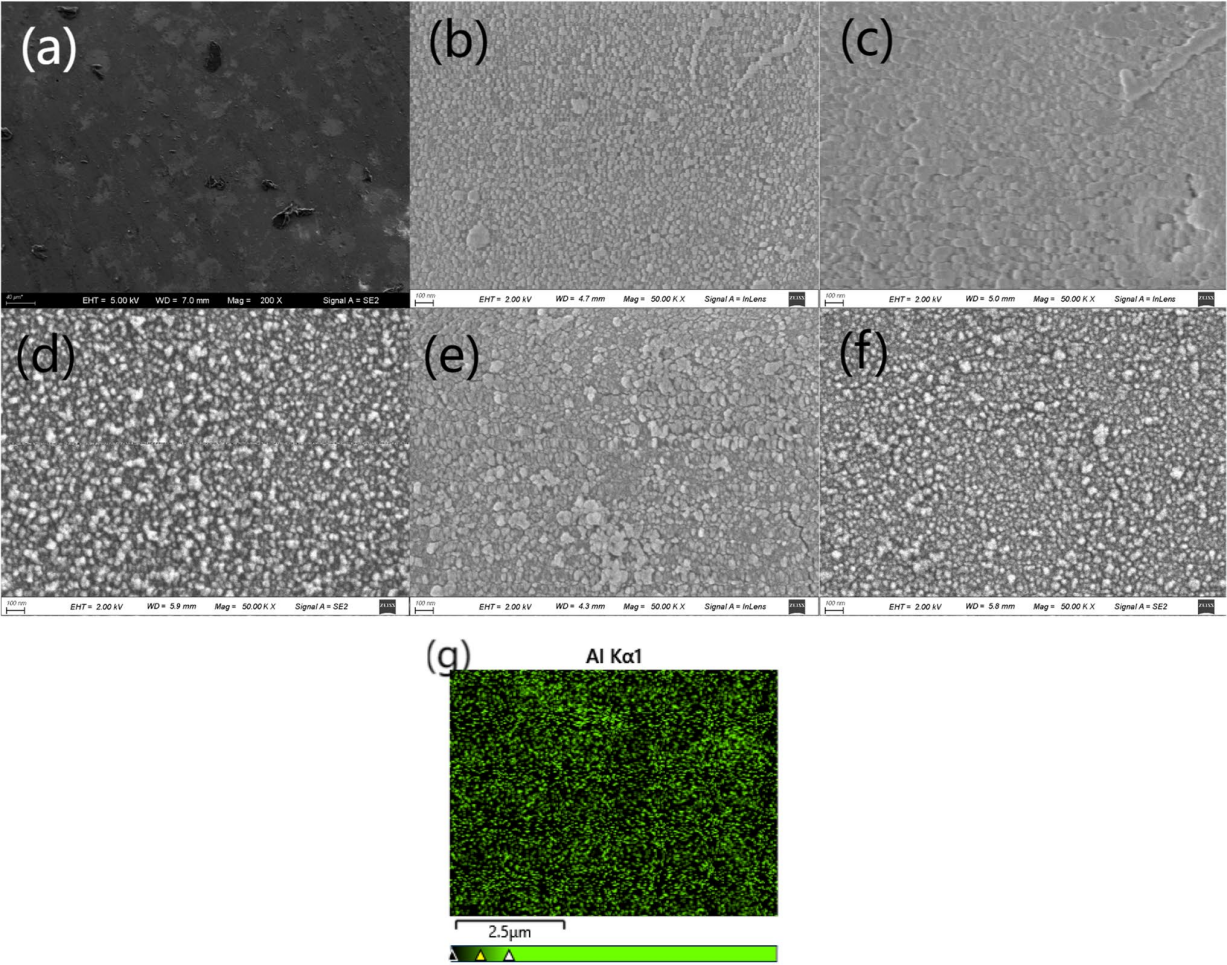
The SEM images of laser ablated Al-coated resin with varying scanning rate and the same 1.6 μm Al coating. **a**, **b** 3 m/s. **c** 2 m/s. **d** 4 m/s. **e** 5 m/s. **f** 6 m/s. **g** EDS mapping for Al element of a representative laser ablated Al-coated resin

Figure 3 presents SEM images of the treated resin with various scanning rates at 1.6 μm Al coating. Figure 3a shows a quasi-periodic micron structure, also a result of the laser scan overlapping. It was worth noting that the black dust present on the surface was due to the sample cutting process. Upon further magnification, irregular nanoparticles were distributed all over the treated resin surfaces, as shown in Fig. 3b–f. The formation of these nanoparticles could be attributed to laser ablation of the Al coating and recast of the ablated material [50]. Oxidation will be inevitable due to reaction between highly active ablated Al and oxygen in the air. Figure 3b–f demonstrates that particle size difference, number of large particles and spacing between particles increased with higher laser scan rate. This suggests slow rate produced densely packed uniformly distributed nanoparticles, which was generally instrumental to hydrophilicity. Figure 3c presents a relatively large amount of pits like dark spots compared with Fig. 3b, which implies laser damage due to the slow scan. The micro-nano structure contributed to increased surface roughness, which may facilitate super hydrophilicity. EDS mapping for Al of a representative laser ablated Al-coated resin was shown in Fig. 3g. It indicates that part of Al coating was ablated away, while the remaining Al was relatively evenly distributed on the sample surface.

### Chemical composition analysis

**Fig. 4:**
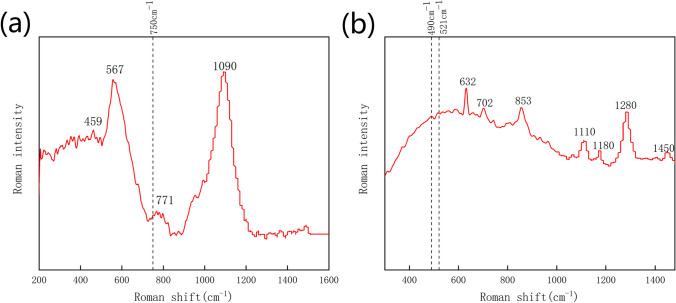
Raman spectroscopy of laser ablated on different substrates with Al thickness of 1.6 μm and scan rate of 3 m/s. **a** Glass. **b** Resin

Figure 4 illustrates Raman spectra of treated glass and resin. The peaks at 459 cm^−1^, 567 cm^−1^, 771 cm^−1^, and 1090 cm^−1^ in Fig. 4a corresponded to the Raman peaks of soda lime glass substrate [51]. The tiny peak at 750 cm^−1^ may be attributed to alumina [52], which may arise from the ablation of Al followed by oxidation and redeposition onto the surface or oxidation of residual Al. Figure 4b presents the Raman spectrum of the resin sample. The weak Raman peak at approximately 490 cm^−1^ and 521 cm^−1^ corresponded to alumina [52], while the remaining Raman peaks originated from the resin substrate. The tiny weak alumina peaks suggestes that a significant portion of the Al film was ablated away during the process. Raman spectra of unprocessed and different scan rates processed glass are shown in Additional file 1: Fig. S1. It discloses almost no difference between the measured samples, suggesting no surface oxidation difference for different samples. Raman spectra differences over different regions of a same sample may be another explanation.

**Fig. 5:**
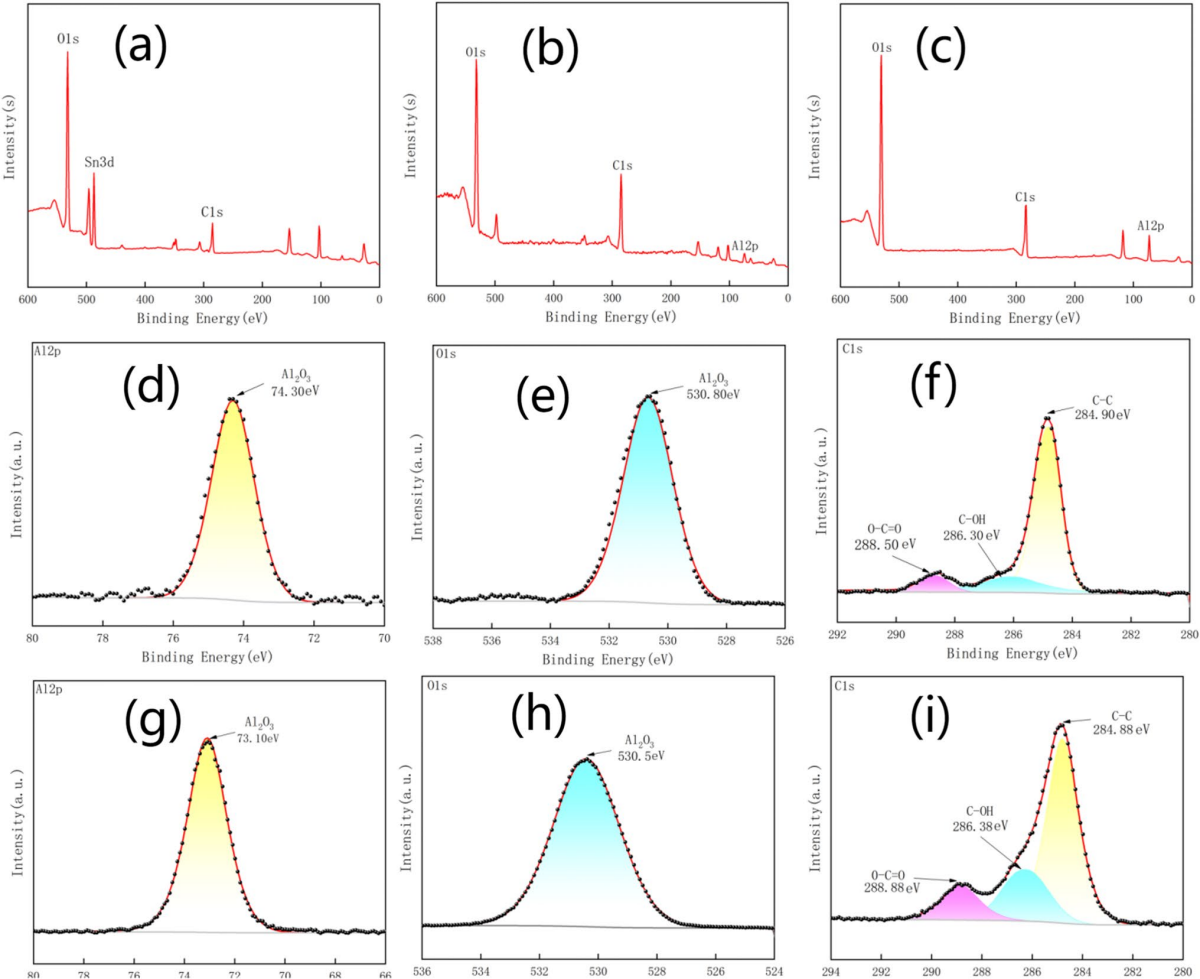
XPS spectra of reference glass and treated samples with Al thickness of 1.6 μm and scan rate of 3 m/s. **a** Reference glass surface, **b** Treated glass surface, **c** Treated resin surface, **d** Al2*p* region in (**b**), **e** O1*s* region in (**b**), **f** C1*s* region in (**b**), **g** Al2*p* region in (**c**), **h** O1*s* region in (**c**), **i** C1*s* region in (**c**)

Figure 5 shows XPS spectra of the reference glass surface, treated glass surface, and treated resin surface. It presents that three main elements C, O, and Al constituted these surfaces, and C1*s* peak, O1*s* peak, and Al2*p* peak were around 284.1 eV, 530.1 eV, and 73.1 eV, respectively. The detection of the Sn peak on the untreated glass was from tin side of glass (float glass inherently contains tin). Carbon was due to adsorption of hydrocarbon organic pollutant from ambient air. The relative strong C peaks on treated substrates compared with reference glass was a result of a few months storage of the treated substrates in air compared to freshly cleaned reference glass before the delivery.

The wettability of a sample is also influenced by its chemical composition [53]. The high-resolution Al2*p* and O1*s* spectra in Fig. 5d, e, g, and h shows that Al and O were mainly bonded in the form of alumina, due to the oxidation during laser ablation. Both Al and O tends to form polar bonds, which are instrumental to superhydrophilicity [54]. The deconvoluted of C1*s* peak was illustrated in Fig. 5f and i. C1*s* was deconvoluted into three peaks at 284.88 eV, 286.38 eV, and 288.88 eV corresponding to C–C (H), C–OH, and O–C=O respectively. C–C (H) was non-polar and adverse to super hydrophilicity. The cumulative content of hydrophilic elements, namely O (37.95%), Al (8.1%), and Si (8.59%), on the glass surface surpassed the content of carbon (C) (40.62%) [39], and the C–C(H) bonds only accounted for 69.99% of the carbon content in the treated glasses. Similarly, the cumulative content of hydrophilic elements O (47.65%) and Al (19.01%) on the resin surface exceeded the content of element C (33.34%). High surface polarity generally results in strong hydrophilicity [39]. This implied that laser treated sample tended to be more hydrophilic than the reference sample.

### Wettability

**Fig. 6:**
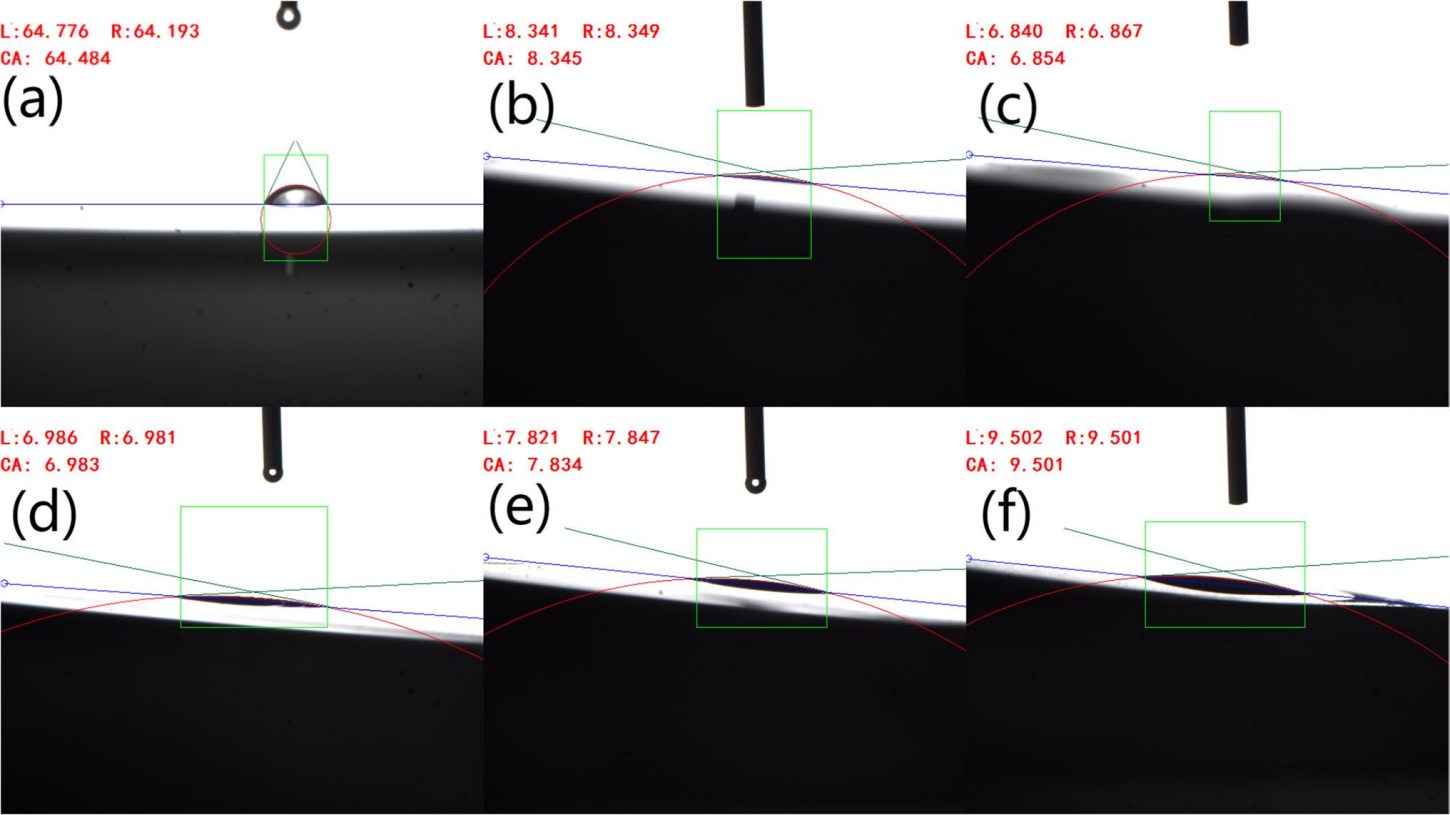
Snapshot contact angle micrograph of treated and reference resin **a** untreated resin and **b**–**f** treated resin with varying laser scan rate and 1.6 μm Al coating: **b** 2 m/s. **c** 3 m/s. **d** 4 m/s. **e** 5 m/s. **f** 6 m/s

One widely employed method to gauge the wettability of a material surface is by measuring the water contact angle [44]. Figure 6 demonstrates contact angles of reference resin and treated resin. Figure 6a indicates the contact angle of reference resin was 64.5º, hydrophilic. Treated resin all exhibited near super-hydrophilic behavior. Figure 6b–f indicates that under the same coating thickness, treated resin exhibited a minimum water contact angle of 6.9° at scan rate of 3 m/s, which is consistent with the roughness in Table 1. This improvement was primarily attributed to the enhanced surface roughness and surface energy achieved through the laser treatment, which was consistent with results in Figs. 2,3,5,6.

**Fig. 7:**
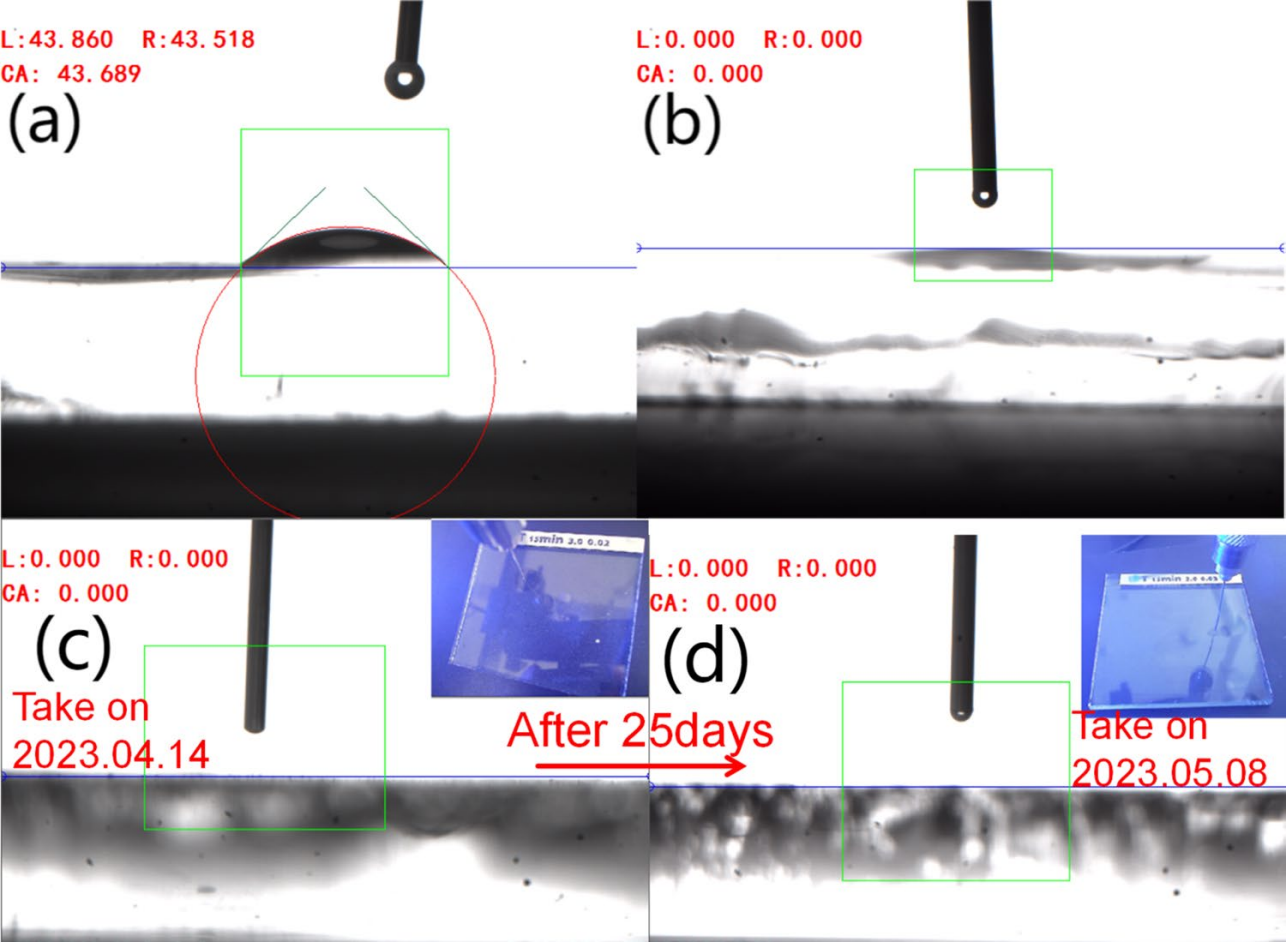
Static contact angle of control glass, laser treated glass with Al thickness of 1.6 μm and scan rate of 3 m/s and contact angle measurement images of the same treated sample over 25 days storage period in the laboratory. **a** Control glass. **b** Treated sample. **c** Contact angle of 0° of the freshly treated sample **d** Contact angle of the same treated sample after 25 days of storage in the laboratory, maintaining 0°. The small window provides a top view of the measurement, displaying surface information, including the mark on the sample

Figure 7a illustrates the water contact angle of the control glass was 44°, indicating hydrophilic behavior. Treated glass achieved 0° contact angle, indicating super hydrophilicity. The contact angles of the treated resin and glass surfaces exhibited notable differences under identical processing conditions, as depicted in Figs. 6c and 7b. This discrepancy can be attributed to the inherent surface energy differences between glass and resin substrates, with the former exhibiting a higher surface energy compared to the latter. Remarkably, Fig. 7c, d demonstrate that the treated glass surface retained a contact angle of 0° for a duration of 25 days, indicating the durable superhydrophilicity of the surface. This durability was attributed to the strong in-organic bonding and stable periodical micro-structure on surface as shown in Figs. 2,5. Al=O in our case may promote the durability since Si=O were found contributing to durable superhydrophilicy [38]. Nanostructures inhibited the airborne organics diffusion. Meanwhile, even under complete coverage of airborne organics on the superhydrophilic surface, the micro-structure still allowed water wetting into the unfilled gaps. Additionally superhydrophilic surface exhibits higher affinity towards water rather than organics, water may penetrate between the surface and the organics in high humid ambient and remove the organics. Therefore, the hierarchical superhydrophilic micro-nano structure played a key role in the durability.

**Fig. 8:**
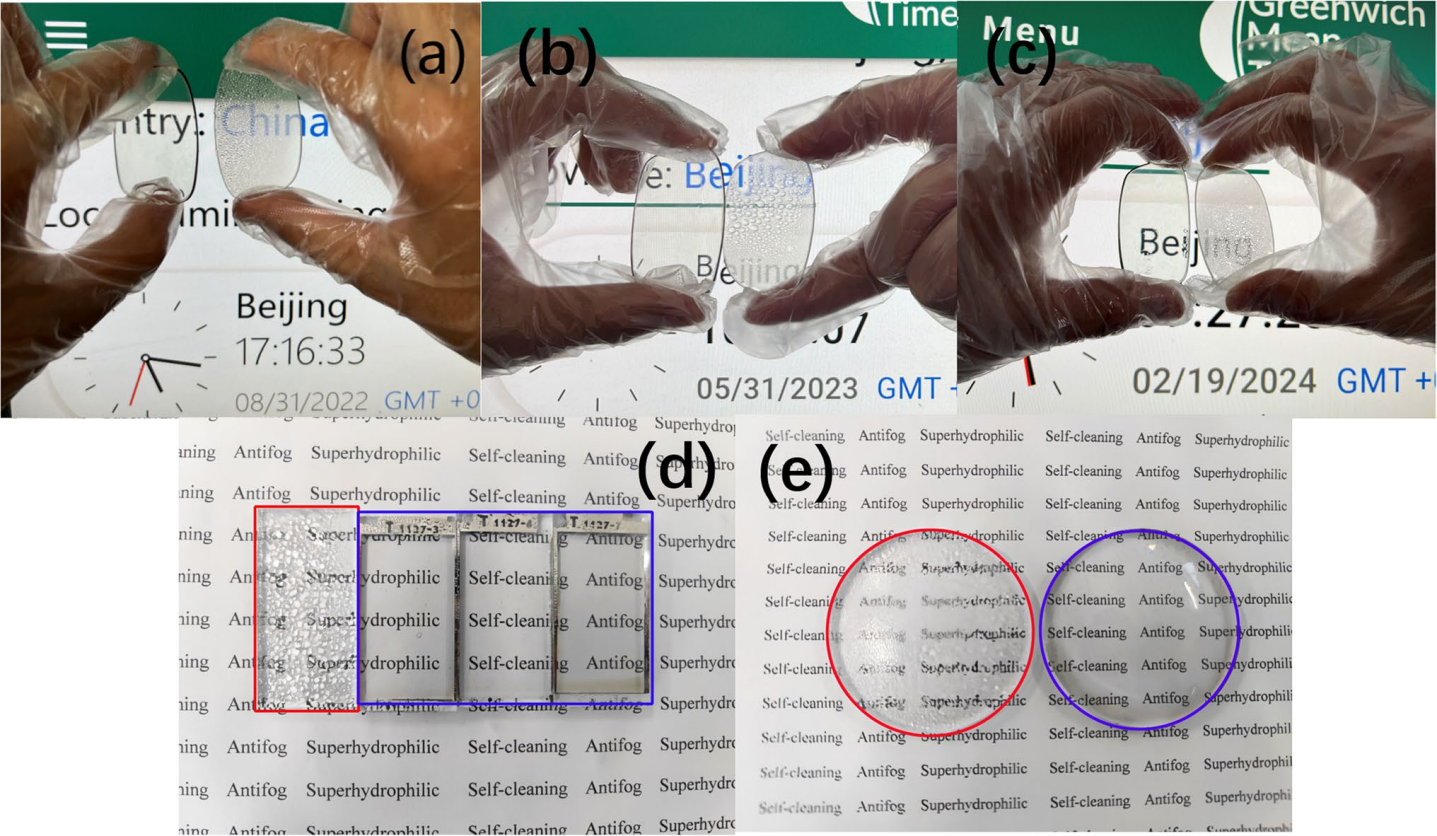
Long-term anti-fog characterization images of a pair of resin glasses (one treated and the other serving as a control) over a storage period of 629 days in laboratory. The treated glass was subjected to a laser processing condition with a 1.6 μm Al coating and a laser scan rate of 3 m/s. The control glass exhibited significant fogging and blurring. The background of the images displays the official website of the Greenwich Observatory, featuring Beijing time. **a** Image acquired on May 31, 2022 **b** Image captured on August 31, 2023. **c** Image taken on Feburary 19 2024. And anti-fog characterization images of untreated reference sample and representative treated samples on different substrates. **d** Reference and treated gla*ss*. **e** Reference and treated resin. (red untreated, blue treated)

Figure 8a–c demonstrates the long-lasting anti-fog characteristic of the treated resin eyeglasses. The eyeglasses were exposed to boiling water vapor, leading to condensation on their surfaces, and the anti-fog effect was evaluated. The findings clearly showed a distinct disparity between the pair of resin glasses. The control sample exhibited blurriness and fogging, causing severe obstruction to light transmission through the resin material. In contrast, the treated sample demonstrated and maintained impressive antifog performance without noticable degradation for a period of 9 months. Even after a storage period of 629 days, the treated sample exhibited anti-fog performance, which was the longest antifog record. The antifog performance degraded substantially after 9 months storage in the lab. This long term antifog performance was originated from robust polar inorganic bonds and stable micro-structure on treated surface as discussed above. Additionally, the micro-structure supported and protected the nano-structure enhancing the durable antifog performance, which was consistent with findings in ref [33]. Figure 8d, e showcases antifog performance of both treated glass and resin samples in comparison to the reference samples. Figure 8d shows that the uncoated glass fogged immediately and became blurry, while the three treated glass slides remained highly transparent. This outcome emphasized the superior anti-fog properties exhibited by the treated glass, highlighting its ability to maintain clarity and ensure the visibility of objects even under challenging foggy conditions. The antifog performance was attributed to the inherent water spreading ability of superhydrophilic surface, which rapidly formed a complete film, eliminating light scattering from droplets.

### Optical characteristics

**Fig. 9:**
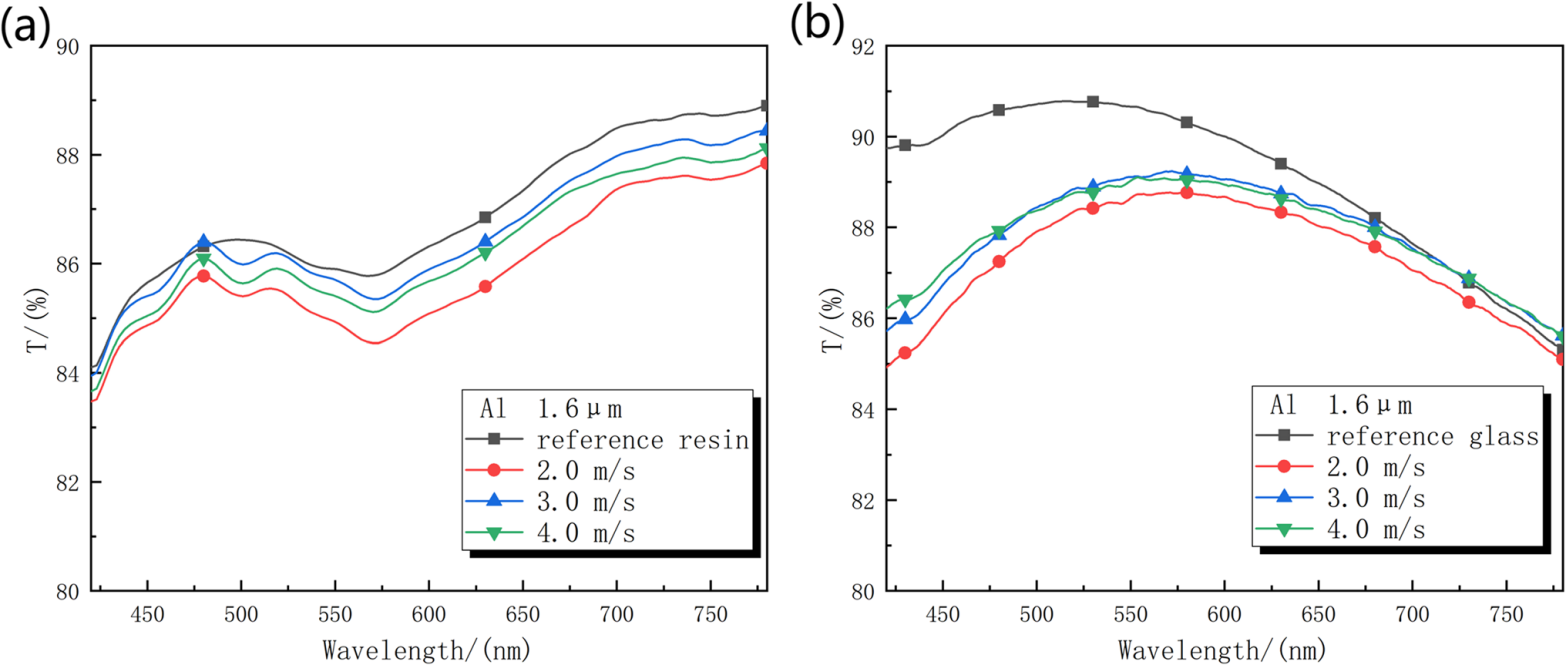
Transmission spectra of laser ablated sample with varying scanning rate on different substrates. **a** Resin. **b** Glass

The transmission of reference samples and treated samples is shown in Fig. 9 and Table 2. It reveals that all resin samples demonstrated comparable absolute transmission values exceeding 85% in the visible spectral range. Figure 9a showed that the treated resin achieved its best transmission at a laser scanning rate of 3 m/s, albeit slightly lower than that of the untreated resin. This might give credit to the densely and uniformly packed nanoparticle structure as shown in Figs. 2a and 3b respectively. Because the nanoparticle structure feature size was smaller than the wavelengths of visible light, which, according to effective medium theory, can mitigate reflection. Conversely, the presence of larger micron structures, as depicted in Fig. 2, may result in scattering losses. Importantly, Fig. 9 suggests that the presence of micron structures undermines the beneficial antireflection effect attributed to the nanoparticle structure, resulting in lower transmission compared to the reference sample. Additionally, Fig. 9b demonstrates that the transmission of treated glass followed similar trend to that of treated resin.

**Table 2:** Average transmission (absolute and relative) of reference sample and processed sample at different scanning speeds. Relative transmission were in the brackets

	Resin (%)	Glass (%)
Reference	86.929 (100%)	89.122 (100%)
2 m/s	85.891 (98.8%)	87.379 (98%)
3 m/s	86.580 (99.6%)	87.876 (98.6%)
4 m/s	86.294 (99.3%)	87.871 (98.6%)

## Conclusions

This article presents a cost effective laser marker ablation of Al coated transparent substrates for creating durable super hydrophilic anti-fog micro-nano structures on their surfaces. An excessively slow scan rate was found to cause over ablation of Al and potentially caused damage to the substrate, which resulted in non-uniformity viewing from the height dimension. While an excessively high scan rate increased nonuniformity and size of the micro-features due to inadequate ablation, which resulted in low roughness values on treated surface. Both non-uniformity and low roughness were adverse to hydrophilicity. Based on the experimental conditions investigated in this study, the optimal process parameters were determined to be a Al coating thickness of 1.6 μm and a laser scan rate of 3 m/s. This process patterned the surface with a hierarchical micro-nano structure leading to an antifog performance record lasting for an impressive duration of 629 days, which is the longest antifog record to date. The antifog performance degraded substantially though after the initial 9 months. The following conclusions were drawn from the study:

1. The water contact angle was substantially reduced significantly from a value of 64.5° for control resin to 6.9° for the optimal treated resin, which exhibited near super-hydrophilic behavior. Likewise, the water contact angle was substantially reduced from 44° for control glass to 0° for the optimal glass, maintaining this exceptional 0° super-hydrophilicity for 25 days. Laser treatment resulted in the formation of alumina on the surface elevating surface energy and generation of micro-nano structures that enhanced surface roughness, thus leading to superhydrophilicity.
2. Practical anti-fog tests validated the outstanding anti-fog performance and durability of the optimal resin sample. The samples exhibited sustained anti-fog performance for an extended period of 629 days. The long lasting hydrophilicity was attributed to the stable micro-nano structure and the strong inorganic bonding on treated surface, namely the hierarchical micro-nano structure characterized by micron hillock-hollow structure as well as widely distributed nanoparticles, and the strong polar bonds with O and Al. Micro-structure facilitated surface wetting, supported and protected the nanostructure, while the nanostructure help inhibit airborne organics diffusion. This hierarchical micro-nano structure thus played a key role in the record long durable antifog performance.

### Supplementary Information

**Additional file 1**. **Fig. S1** Raman spectroscopy of unprocessed and processed glass with different scan rates. (a) reference (b) 2 m/s (c) 4 m/s (d) 5 m/s (e) 6 m/s.

## Data Availability

The data that support the findings of this study are available from the corresponding author upon reasonable request.
