# Supplementary material for: Durable anti-fog micro-nano structures fabricated by laser ablation of aluminum film on resin/glass
Source: Discov Nano. 2024 Mar 26;19(1):58. doi: 10.1186/s11671-024-03993-y (PMC10965884; doi:10.1186/s11671-024-03993-y)
Supplement: Supplementary file 1 — Additional file 1. Fig. S1 Raman spectroscopy of unprocessed and processed glass with different scan rates. (a) reference (b) 2 m/s (c) 4 m/s (d) 5 m/s (e) 6 m/s. [file 11671_2024_3993_MOESM1_ESM.docx]

SUPPORTING INFORMATION

Durable Anti-Fog Micro-Nano Structures Fabricated by Laser Ablation of Aluminum Film on Resin/Glass

Hongtao Cui*, Chao Teng, Xinyi Xie, Xiaowen Qi

Department of Materials Science, School of Civil Engineering, Qingdao University of Technology, Qingdao 266520, China

Correspondence*: cuihongtao88@gmail.com


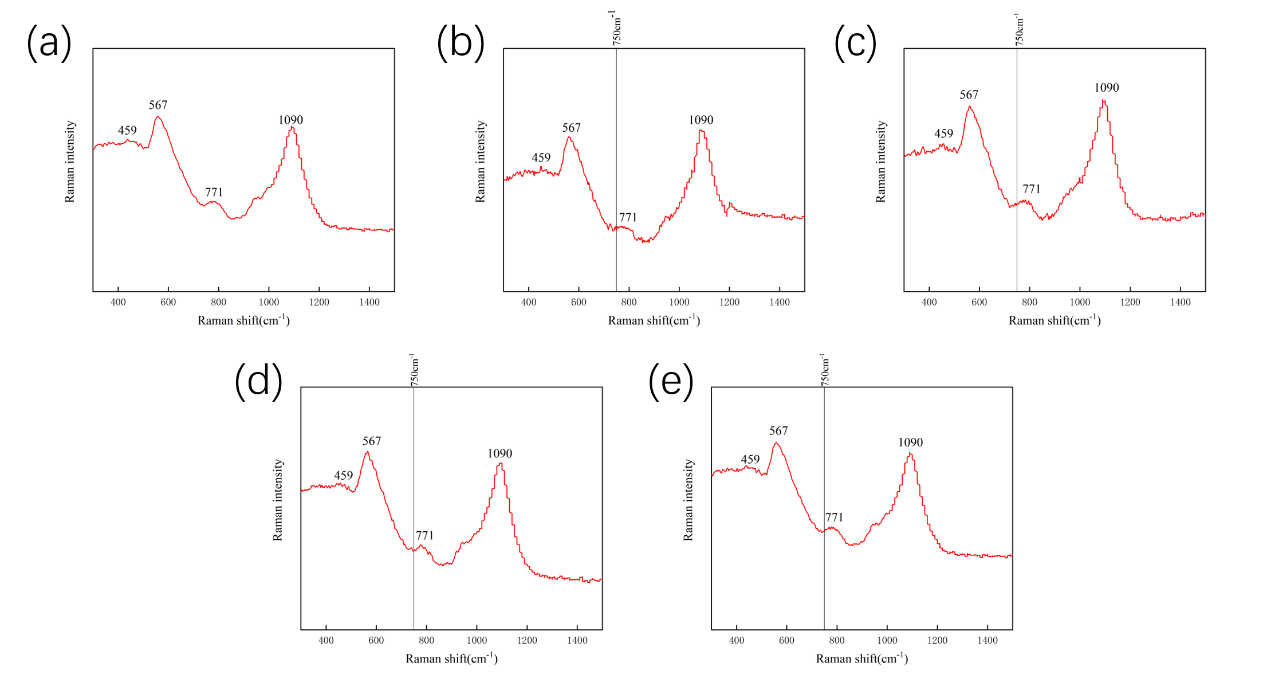


Fig.S1 Raman spectroscopy of unprocessed and processed glass with different scan rates. (a) reference (b) 2 m/s (c) 4 m/s (d) 5m/s (e) 6m/s
